# Pure White Cell Aplasia, an Exceedingly Rare Complication of a Thymoma, and Immune Reconstitution Following Bone Marrow Recovery

**DOI:** 10.3390/hematolrep17020014

**Published:** 2025-03-21

**Authors:** Giby V. George, J. C. Uy, John L. Mariano, Marisa Jacob-Leonce, Chauncey R. Syposs

**Affiliations:** 1 Department of Pathology and Laboratory Medicine, University of Rochester Medical Center, Rochester, NY 14642, USAjann_uy@urmc.rochester.edu (J.C.U.);; 2 Department of Hematology and Oncology, University of Rochester Medical Center, Rochester, NY 14642, USA

**Keywords:** thymoma, pure white cell aplasia, immune reconstitution

## Abstract

Background: Thymoma-associated pure white cell aplasia (PWCA), characterized by agranulocytosis with absent myeloid precursors in the bone marrow in the setting of preserved erythropoiesis and megakaryopoiesis, is exceedingly rare, with only a few cases reported in the literature. We present a case of type-B2-thymoma-associated PWCA and immune reconstitution following marrow recovery. Case Presentation: A 75-year-old woman was incidentally found to have a concomitant mediastinal mass and peripheral leukopenia with absent granulocytes and monocytes. Bone marrow assessment was notable for a hypocellular marrow (<10%) with absent granulopoiesis and monopoiesis. Chest CT demonstrated a large lobulated anterior mediastinal mass, for which the patient underwent a video-assisted thoracoscopic thymectomy. Pathological evaluation of the mediastinal mass specimen revealed a type B2 thymoma. A tentative diagnosis of thymoma-associated PWCA was made, and the patient was started on cyclosporine/granulocyte-colony stimulating factor (G-CSF)/filgrastim therapy. Despite promising marrow recovery, she developed several comorbidities and had a leukemoid reaction, provoking concern for immune reconstitution following prolonged neutropenia and subsequent treatment. She passed away on post-operative day 15, and the results of a post-mortem bone marrow examination were consistent with granulocytic hyperplasia. Conclusions: This case of thymoma-associated PWCA heightens awareness regarding this entity, providing a note of caution regarding the possibility of immune reconstitution following treatment and marrow recovery.

## 1. Background

Thymomas, thymic carcinomas, and thymic neuroendocrine neoplasms constitute thymic epithelial tumors [1]. Thymomas are sub-classified (i.e., Type A, AB, B1, B2, and B3) based on the morphology and neoplastic epithelial component [1]. Though uncommon, accounting for less than 1% of all neoplasms [2], thymomas and thymic carcinomas may be associated with various paraneoplastic manifestations [1]. Such disorders include those affecting the following systems: neuromuscular, hematologic, collagen/autoimmune, immune deficiency, endocrine, dermatologic, and others [1]. Of these, the five most commonly reported paraneoplastic syndromes in association with thymomas include myasthenia gravis (MG) (reported in up to 50% of cases), limbic encephalopathy, pure red cell aplasia (PRCA) (identified in nearly 5% of cases), Good Syndrome/hypogammaglobulinemia (observed in 6–11% of cases), and lichen planus [1,3,4]. Thymoma-associated pure white cell aplasia (PWCA), characterized by agranulocytosis and extreme monopenia with absent myeloid precursors in the bone marrow in the setting of preserved erythropoiesis and megakaryopoiesis, is exceedingly rare, with only a few cases reported in the literature [3,4,5,6,7,8,9]. In the setting of Good’s syndrome-associated thymoma, PWCA only affects approximately 1.1% of patients [4]. Additionally, most cases of PWCA have been reported in association with types A and AB thymomas [4,9].

Here, we present a case of thymoma (type B2)-associated PWCA and immune reconstitution following marrow recovery. We also provide an overview of thymoma-associated PWCA along with a review of the literature.

## 2. Case Presentation

**Figure 1:**
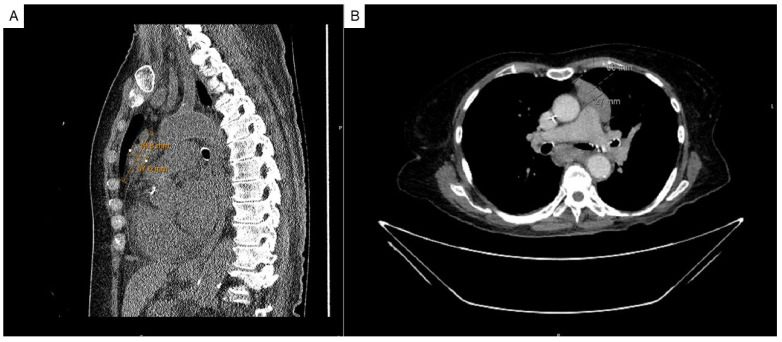
(**A**) Sagittal and (**B**) axial views of the CT scan of the chest (without contrast), demonstrating a large, lobulated anterior mediastinal mass measuring 6.6 × 3.5 × 7.5 cm abutting the left anterolateral aspects of the ascending aorta and main pulmonary artery.

A 75-year-old female with a medical history significant for Hashimoto’s thyroiditis and Sjogren’s syndrome presented for an evaluation of a mediastinal mass noticed incidentally on a routine chest X-ray. A CT scan of her chest revealed a large lobulated anterior mediastinal mass measuring 6.6 × 3.5 × 7.5 cm, abutting the left anterolateral aspect of the ascending aorta and main pulmonary artery (Figure 1A,B).

**Figure 2:**
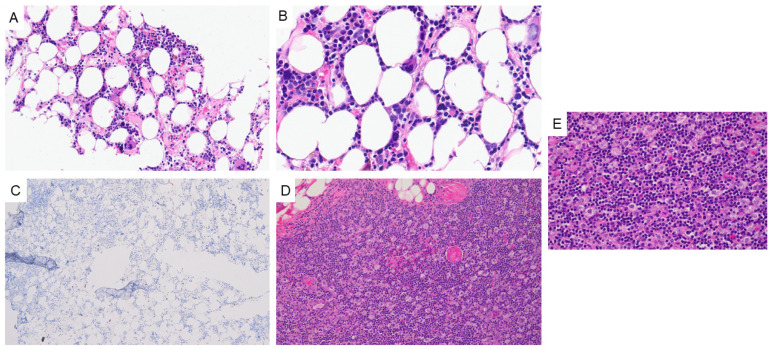
Bone marrow evaluation revealed a hypocellular marrow with preserved erythropoiesis and megakaryopoiesis and absent granulopoiesis and monopoiesis as evidenced by the negative expression of myeloperoxidase (MPO): ((**A**) 20x, (**B**) 40x, and (**C**) 10x). Histopathologic evaluation of the resected mediastinal mass specimen revealed a type B2 thymoma (stage pT1aN0, AJCC 8th edition) ((**D**) 20x and (**E**) 40x).

Pathology evaluation of a core needle biopsy specimen showed a scant fragment of hyperplastic thymic tissue, favoring primary thymic neoplasia over reactive thymic hyperplasia. Flow cytometry analysis proved unremarkable, showing normal T-cells in multiple stages of maturation. Laboratory investigations and peripheral blood examination revealed leukopenia (absolute lymphocyte count: 0.3 × 10^3^/µL, normal range: 0.9–3.8 × 10^3^/µL) with absent neutrophils, eosinophils, basophils, and monocytes and preserved erythropoiesis (hemoglobin: 13.2 g/dL, normal range: 11.2–16 g/dL) and thrombocytopoiesis (platelet count: 250 × 10^3^/µL, normal range: 150–450 × 10^3^/µL). Bone marrow evaluation was notable for a significantly hypocellular marrow with absent granulopoiesis and monopoiesis (Figure 2A–C).

Concurrent cytogenetics (karyotype and interphase fluorescence in situ hybridization (FISH) using acute myeloid leukemia [AML] probes) and molecular (targeted DNA-based next-generation sequencing [NGS]) workup was negative for any cytogenetic or molecular aberrations that might have suggested an underlying primary bone marrow pathology. Subsequent histopathologic evaluation of the resected mediastinal mass specimen showed a type B2 thymoma (stage pT1aN0, AJCC 8th edition) (Figure 2D,E). In light of these results and the bone marrow findings (i.e., absent granulopoiesis and monopoiesis), a tentative diagnosis of thymoma-associated PWCA was made, although neutropenia secondary to the patient’s autoimmune conditions could not entirely be excluded.

**Figure 3:**
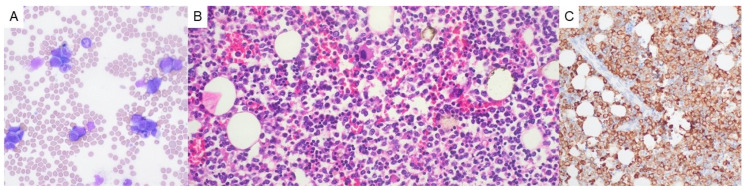
(**A**) Peripheral blood examination (50x oil lens objective) demonstrating a leukemoid reaction with significant left shift. (**B**) Pathologic evaluation of the bone marrow demonstrated hypercellularity, with >70% of cells showing MPO positivity (**C**) with maturing granulocytic forms (both at 40x lens objective).

The patient was started on cyclosporine, granulocyte colony-stimulating factor (G-CSF), and eventually filgrastim (480 mcg daily for a total of 12 doses), with the last dose stopped upon absolute neutrophil count recovery (from 0 × 10^3^/µL at the start of treatment to 200 × 10^3^/µL post-treatment). She received no steroid therapy over this period of time. Despite neutropenic prophylaxis with ciprofloxacin, she developed *S. mitis* bacteremia, for which she was treated with amoxicillin, cefepime, and vancomycin. She also developed right-subclavian-vein thrombosis and cerebral venous sinus thrombosis in the setting of antiphospholipid antibody syndrome (positive for lupus anticoagulant, anticardiolipin, and beta-2 glycoprotein antibodies). She subsequently developed a leukemoid reaction (Figure 3A), in which abundant marrow elements (both myeloid and erythroid) could be observed in the peripheral blood at all stages of maturation, likely secondary to immune reconstitution following prolonged neutropenia and cyclosporine/G-CSF/filgrastim therapy or an exaggerated response to an underlying, occult infection. To rule out an underlying myeloproliferative process such as chronic myeloid leukemia, FISH for *BCR::ABL* was performed, which proved negative.

The neurology department was consulted for progressive encephalopathy and worsening muscle weakness of unknown etiology, provoking concern for myasthenia gravis. An encephalopathy workup and acetylcholine receptor antibody testing, however, were unremarkable. No new infections were identified following her initial bacteremia, as evidenced by negative blood cultures. Regardless, shock (likely distributive) progressed despite supportive management, dexamethasone/tocilizumab therapy for immune reconstitution, and continuous renal replacement therapy (CRRT) for critical acidemia. Given the patient’s rapid deterioration, she was transitioned to comfort care.

Post-mortem external examination was notable for diffuse anasarca. Internal examination revealed bilateral pleural effusions, pericardial effusion, and ascites (all serous fluid in nature with no evidence of cellular infiltration), as well as multifocal clusters of pitted, red-brown submucosal nodules of the intestinal tract (up to 1 cm in size), which was consistent with lymphocytic colitis on pathologic evaluation. The bone marrow was hypercellular, with >70% of cells showing MPO positivity with maturing granulocytic forms (Figure 3B,C). Pathologic evaluation of the thymoma bed showed fibroadipose tissue with scattered lymphoid aggregates with no evidence of residual thymoma.

## 3. Discussion and Conclusions

Unlike thymoma-associated PRCA, PWCA in the setting of thymomas (and thymic carcinomas) [10] is incredibly rare, with only a few cases reported in the literature [3,4,5,6,7,8,9]. PWCA is characterized by peripheral blood agranulocytosis and bone marrow granulocytic aplasia with preserved erythropoiesis and megakaryopoiesis. It has been reported in association with various drugs (antibiotics, antithyroid drugs, clozapine, etc.), infectious diseases (viral infections, Lyme disease, fungal infections, sepsis, etc.), and autoimmune processes (rheumatoid arthritis, systemic lupus erythematosus, etc.) [3,11]. Although the pathophysiology of thymoma-associated PWCA is unclear, autoimmunity in this setting may arise from combined dysregulation of the cellular and humoral immune systems (i.e., autoreactive T-cells promote B-cells to produce auto-antibodies) [3]. Uy et al. raise the possibility of dysregulated cytokine production by neoplastic thymic stromal cells, which promote the expansion of thymic cells and precursor B-cells in the bone marrow [4]. Other theories include autoreactive T-cells arising from immature, neoplastic T-cells and genetic alterations that might predispose one to autoimmunity (e.g., decreased HLA-DR expression) [3,4]. Interestingly, calcineurin inhibitors, such as cyclosporine, which interfere with the production of interleukin (IL-2) and other cytokines [12], are effective in the treatment of both PRCA and PWCA, supporting a key role for T-cell mediated autoimmunity [3]. Thymectomy, which is the standard treatment for patients with thymoma, may also aid in the resolution of autoimmune manifestations by removing the source of neoplastic, autoreactive T-cells [3].

Given the rarity of this phenomenon, the medical management for thymoma-associated PWCA has not yet been established [3]. However, patients with thymoma-associated PWCA are at an increased risk of experiencing fatal neutropenic sepsis, necessitating prompt confirmation of agranulocytosis via bone marrow biopsy to enable immediate therapeutic intervention. Combined surgical/immunosuppressive treatment has demonstrated improvement in a few cases [3,7]. In a review of the literature, Lopez et al. found that while G-CSF, IVIg, and plasmapheresis were not associated with much efficacy in this setting, immunosuppressants, namely, cyclosporine and, to a lesser extent, other agents such as azathioprine, cyclophosphamide, alemtuzumab, and corticosteroids, were effective in promoting marrow recovery [3]. In a more recent report, Yang et al. describe the need for long-term continuation of cyclosporine for granulocytic response, following the acquisition of initial marrow results and a subsequent drop in counts following cessation of immunosuppressive therapy [8]. Likewise, Youssef et al. describe the case of a type B1 thymoma, in which the patient developed PWCA and immune thrombocytopenia following thymectomy [9]. Despite combined therapy with IVIg, G-CSF, prednisone, cyclosporine, and eltrombopag, however, the patient died from sepsis and multiorgan failure [9]. Interestingly, Becker and colleagues reported successful neutrophil recovery with sunitinib in the treatment of a patient with a type B2 thymoma based on a few clinical trials [5,13,14,15].

Immune reconstitution inflammatory syndrome (IRIS) (also referred to as immune reconstitution syndrome [IRS] and other terminologies) refers to the reconstitution of pathogen-specific host responses and is characterized by a dramatic and exaggerated dysregulated immune response often involving multiple organ systems [16]. IRS has been well-documented in the setting of HIV-infected individuals following the initiation of highly active antiretroviral therapy (HAART) and during the treatment of associated infections, such as tuberculosis and *M. avium* complex infections [16]. Sun and Singh also describe the development of IRS in transplant recipients, neutropenic patients, and patients receiving tumor necrosis factor (TNF)-α inhibitors, in addition to other scenarios [16]. In our case, the patient began G-CSF therapy nine days prior to thymectomy without granulocytic recovery. G-CSF was continued post-thymectomy, and cyclosporine and filgrastim were added, after which the patient showed granulocytic recovery by post-operative day 12. However, on post-op day 14 she developed a leukemoid reaction and several comorbidities. These clinicopathologic findings provoked concern for either immune reconstitution following prolonged neutropenia and subsequent treatment. To the best of our knowledge, this phenomenon has not yet been reported in the context of thymoma-associated PWCA treatment. Alternatively, her symptomology may have stemmed from an exaggerated response to an underlying, occult infection, although this is less likely in light of her negative blood cultures following treatment for her initial bacteremia.

In conclusion, our case highlights the difficulty in the diagnosis and treatment of thymoma-associated PWCA. Although treatment with G-CSF, cyclosporine, and filgrastim enabled granulocytic recovery in our patient’s case, several comorbidities and a leukemoid reaction provoked concern for immune reconstitution following prolonged neutropenia and directed treatment. Although further studies are needed to weigh the benefits and timing of treatment for PWCA, the over-administration of G-CSF and/or filgrastim and the development of an infection secondary to treatment may lead to immune reconstitution. Thus, a high index of suspicion is needed during the workup of a thymoma, which may present with a broad spectrum of paraneoplastic manifestations, including PWCA.

## Data Availability

Data are contained within the article.

## References

[1] Marx A.D.F., Maron E.M., WHO Classification of Tumours Editorial Board (2021). Tumours of the thymus. Thoracic Tumours [Internet].

[2] Weis C.A., Yao X., Deng Y., Detterbeck F.C., Marino M., Nicholson A.G., Huang J., Strobel P., Antonicelli A., Marx A. (2015). The impact of thymoma histotype on prognosis in a worldwide database. J. Thorac. Oncol..

[3] Cespedes Lopez R., Amutio Diez E., Martin Martitegui X., Balerdi Malcorra A., Insunza Oleaga L., Arzuaga-Mendez J., Moreno Gamiz M., Saiz Camin M., Aberasturi Plata Y., Garcia-Ruiz J.C. (2022). Pure white cell aplasia an exceptional condition in the immunological conundrum of thymomas: Responses to immunosuppression and literature review. Clin. Case Rep..

[4] Uy K., Levin E., Mroz P., Li F., Shah S. (2019). A Rare Complication of Thymoma: Pure White Cell Aplasia in Good’s Syndrome. Case Rep. Hematol..

[5] Becker H., Auman K., Claus R., von Bubnoff N., Sachs U.J., Waller C.F. (2017). Sunitinib in the Treatment of Thymoma and Associated Autoimmune Neutropenia. JCO Precis. Oncol..

[6] Fumeaux Z., Beris P., Borisch B., Sarasin F.P., Roosnek E., Dayer J.M., Chizzolini C. (2003). Complete remission of pure white cell aplasia associated with thymoma, autoimmune thyroiditis and type 1 diabetes. Eur. J. Haematol..

[7] Kobayashi Y., Ando K., Hata T., Imaizumi Y., Nagai K., Kamijyo R., Katoh T., Taguchi J., Itonaga H., Sato S. (2019). Complete remission of pure white cell aplasia associated with thymoma after thymectomy and cyclosporine administration. Int. J. Hematol..

[8] Yang Y., Chen C., Zheng B., Fan L., Chen X., Hu M. (2024). Pure white cell aplasia before and after thymectomy in the rare conundrum of thymoma: A case report and review of the literature. Medicine.

[9] Youssef M., Stratton T.W., Gallant R.C., Young C., Li D.Y., Piran S. (2022). Pure White Cell Aplasia and Immune Thrombocytopenia after Thymoma Resection: A Case Report and Review of the Literature. Case Rep. Hematol..

[10] Desai P.C., Jones P. (2013). Pure white cell aplasia in a patient with thymic carcinoma. Blood.

[11] Curtis B.R. (2017). Non-chemotherapy drug-induced neutropenia: Key points to manage the challenges. Hematol. Am. Soc. Hematol. Educ. Program.

[12] Tsuda K., Yamanaka K., Kitagawa H., Akeda T., Naka M., Niwa K., Nakanishi T., Kakeda M., Gabazza E.C., Mizutani H. (2012). Calcineurin inhibitors suppress cytokine production from memory T cells and differentiation of naive T cells into cytokine-producing mature T cells. PLoS ONE.

[13] Proto C., Manglaviti S., Lo Russo G., Musca M., Galli G., Imbimbo M., Perrino M., Cordua N., Rulli E., Ballatore Z. (2023). STYLE (NCT03449173): A Phase 2 Trial of Sunitinib in Patients With Type B3 Thymoma or Thymic Carcinoma in Second and Further Lines. J. Thorac. Oncol..

[14] Thomas A., Rajan A., Berman A., Tomita Y., Brzezniak C., Lee M.J., Lee S., Ling A., Spittler A.J., Carter C.A. (2015). Sunitinib in patients with chemotherapy-refractory thymoma and thymic carcinoma: An open-label phase 2 trial. Lancet Oncol..

[15] Remon J., Girard N., Mazieres J., Dansin E., Pichon E., Greillier L., Dubos C., Lindsay C.R., Besse B. (2016). Sunitinib in patients with advanced thymic malignancies: Cohort from the French RYTHMIC network. Lung Cancer.

[16] Sun H.Y., Singh N. (2009). Immune reconstitution inflammatory syndrome in non-HIV immunocompromised patients. Curr. Opin. Infect. Dis..

